# Host-Microbe Interactions in *Caenorhabditis elegans*

**DOI:** 10.1155/2013/356451

**Published:** 2013-08-01

**Authors:** Rui Zhang, Aixin Hou

**Affiliations:** Department of Environmental Sciences, School of the Coast and Environment, Louisiana State University, Baton Rouge, LA 70803, USA

## Abstract

A good understanding of how microbes interact with hosts has a direct bearing on our capability of fighting infectious microbial pathogens and making good use of beneficial ones. Among the model organisms used to study reciprocal actions among microbes and hosts, *C. elegans* may be the most advantageous in the context of its unique attributes such as the short life cycle, easiness of laboratory maintenance, and the availability of different genetic mutants. This review summarizes the recent advances in understanding host-microbe interactions in *C. elegans*. Although these investigations have greatly enhanced our understanding of *C. elegans*-microbe relationships, all but one of them involve only one or few microbial species. We argue here that more research is needed for exploring the evolution and establishment of a complex microbial community in the worm's intestine and its interaction with the host.

## 1. Introduction

Host-microbe symbiosis exists in almost all animals, and the symbiotic bacteria can be profitable, harmful, or of no effect to the host. For example, the harmless *Escherichia coli *strains commonly found in organismal intestine are a normal part of the gut flora and can advantage their hosts by producing vitamin K [1] and by keeping pathogenic bacteria from colonizing the intestine [2, 3]. By contrast, some others like *E. coli* strain O26 can cause diseases in its hosts [4]. The interactions between host and microbe form complicated networks. Understanding these interactions can help us effectively cure the diseases caused by pathogenic microbes and promote good health in animals by benign microbes. A number of model organisms, for example, *C. elegans*, *Saccharomyces cerevisiae*, *Drosophila melanogaster*, Arabidopsis, zebrafish, and mice, have been used to study the mechanisms involved in host-microbe interactions. Among these organisms, *C. elegans* has its unique characteristics that can be regarded as its advantages being a model animal.

The nematode *C. elegans* is a free living, multicellular invertebrate. Its two original strains were isolated from soil in France [5] and mushroom compost in England [6] more than a half-century ago. Naturally *C. elegans* lives on microbes including bacteria and fungi, and it is also a natural host of some pathogenic microorganisms such as the Gram-negative bacterium *Microbacterium nematophilum*, the fungus *Drechmeria coniospora*, the microsporidian parasite *Nematocida parisii*, and the Orsay virus [7, 8]. Major attributes of *C. elegans* as a model include its tractability to be easily maintained in the laboratory, the availability of a great number of genetic mutants, its facility for gene downregulation, and the short life cycle of approximately 2 to 3 weeks. *C. elegans* has been used as a model organism since 1965 [9, 10] in a broad range of research areas, including RNA interference, aging mechanism, regulation of fat storage, host-pathogen relationships, pyrimidine biosynthesis pathway [11], apoptosis, DNA damage responses, genotoxic stress [12], exploration of useful food constituents and drug candidates [13], and identification of new antioxidant probiotic strains for potential use in humans [14]. We review here various interactions between *C. elegans* and microbes, with a focus on the recent findings in understanding the mechanisms of this host-microbe relationship.

## 2. Interactions between Microbial Pathogens and *C. elegans*

A wide variety of bacterial pathogens, as well as several fungi, can kill *C. elegans* or produce nonlethal disease symptoms in the worm. Notably, these microbes include some well-known human pathogens such as Gram-negative bacteria of genera *Burkholderia*, *Pseudomonas*, *Salmonella*, *Serratia*, and *Yersinia*; Gram-positive bacteria *Enterococcus*, *Staphylococcus*, and *Streptococcus*; and the fungus *Cryptococcus neoformans*. Next, we summarize the mechanisms involved in the interplay of *C. elegans* with its microbial pathogens.

### 2.1. Tactics Pathogens Apply to Injure *C. elegans*

Usually, microbial pathogens colonize the *C. elegans* intestine and decrease its lifespan. Some of them adhere to the nematode cuticle, while others produce toxins that kill *C. elegans* without a need for the live bacterial cells to directly contact with the worm [15]. The following (presented chronologically) are some examples of major tactics that pathogenic microbes apply to injure *C. elegans*.

- *Salmonella enterica* serovar Typhimurium is found to be virulent in *C. elegans* through modulation of its virulence by DNA adenine methyltransferase (DAM) [16].
- A coryneform bacterium named *Microbacterium nematophilum* can colonize the rectum of infected worms and cause localized swelling, coprostasis, and retardant growth [17].
- *Listeria monocytogenes* can infect *C. elegans* extracellularly, leading to its death by bacterial accumulation in the worm intestine [18].
- Direct interaction between live nontyphoidal salmonellae (NTS), a bacterium that causes gastroenteritis worldwide, and *C. elegans* is necessary for the worm's lethality [19].
- *Yersinia enterocolitica* strains can kill *C. elegans*, and the tcaA gene that encodes an insecticidal toxin plays a great role in the nematocidal activity [20].
- In soil, in order to prevent themselves from being predated by bacterivorous nematodes, many bacteria have evolved defense mechanisms such as releasing toxic molecules. For example, the extracellular secondary metabolites that are produced by the model soil bacterium *Pseudomonas fluorescens* function as a defense strategy against bacterivorous nematodes [21].
- *Cronobacter sakazakii*, a human pathogen, can infect *C. elegans* at an infection rate that depends on the bacterial accumulation inside the host and induce the antimicrobial genes in *C. elegans* [22].
- *Shigella flexneri*, the causative agent of bacillary dysentery, can kill *C. elegans*. The bacterial virulence genes and the candidate antimicrobial genes of the host are kinetically regulated in *C. elegans* during infection [23].
- Bacterial mucoid strains synthesize exopolysaccharide matrix to avoid the induction of NPR-1-dependent behaviors of *C. elegans* so as to inhibit specific host responses to microbes [24].
- The yeast form of *Candida albicans* can cause an intestinal infection in *C. elegans*, whereas heat-killed yeast is innocuous. This indicates that the host response to *C. albicans* is largely mediated by pattern recognition, that is, pathogen-associated molecular patterns or PAMPs, which are a group of conserved microbial molecules. Also, it has been found that in response to fungal pathogens, *C. elegans* selectively represses the transcription of antibacterial immune effectors. That is to say, nematodes selectively mount specific antifungal defenses at the expense of antibacterial responses [25].
- Different strains of *Burkholderia pseudomallei*, the causative agent of melioidosis that can cause considerable damage in animals, have diverse ability to kill *C. elegans*. Its virulence depends on a junction of genetic and environmental factors. *C. elegans* requires proliferating *B. pseudomallei* to consecutively produce toxins to regulate thorough killing [26].
- *Vibrio cholerae* cytolysin (VCC) is reported as a major virulence factor, which invokes multifarious immune response related genes during *V. cholerae* infection in *C. elegans* [27].
- Three lactic bacteria strains, *Lactobacillus salivarius*, *Lactobacillus reuteri*, and *Pediococcus acidilactici*, have been found to hinder the worm's development and growth, obstruct the germ cells growth, and induce the gene expressions involved in pathogen response [28].

Additionally, it is worth noting that the virulence of microbes can be impacted by preinfection of *C. elegans* with other pathogens. For instance, preinfection with pathogen *Staphylococcus aureus* increases the vulnerability of the *C. elegans* host by perverting its immune system, which then allows the opportunistic pathogen *Proteus mirabilis* to be pathogenic to this host [29]. Also, pore-forming toxins (PFTs) are important bacterial virulence factors and essential for the pathogenesis of many Gram-positive and Gram-negative bacteria. They act by poking holes in the plasma membrane of cells. Besides, horizontal gene transfer (HGT) between bacteria occurs in the intestinal tract of their animal hosts and facilitates both virulence and antibiotic resistance [30]. Last, it has been discovered that Orsay virus can infect *C. elegans* and cause abnormal morphologies of *C. elegans* intestinal cells; the mechanism underneath this type of infection has not been revealed yet [7].

### 2.2. Tactics Employed by *C. elegans* to Battle against Pathogens

A number of strategies are known to be used by *C. elegans* to battle against pathogens. First,* C. elegans* owns mechanisms to avoid certain pathogens. For example, *C. elegans* specifically avoids some strains of *Serratia* appertaining to their production of the cyclic lipodepsipentapeptide serrawettin W2. This voidance behavior requires G protein signaling pathways and the sole *C. elegans* Toll-like receptor TOL-1 [31]. The hypothesis is further supported by the finding that pathogen recognition through Toll-like receptors (TLRs) is crucial for *C. elegans* to launch an appropriate immune response against microorganisms. A study [32] has observed that the tol-1 mutated *C. elegans* can be killed by the human pathogen *Salmonella enterica* by causing a marked pharyngeal invasion. It also has reported that TOL-1 in *C. elegans* is required for the normal expression of two pharyngeal-expressed genes, a defensing like molecule ABF-2 and a heat-shock protein 16.41, which is essential for *C. elegans* immunity. These findings confirm that TOL-1 has a direct effect on defense response of *C. elegans* to some pathogenic microbes. Another bolstering example is that *C. elegans* can avoid cultures and culture supernatants of pathogenic bacterium *Staphylococcus aureus* by recognizing secretory molecules including toxic shock syndrome toxin 1 (TSST-1) and staphylococcal enterotoxin C (SEC). This avoidance is found to be dependent on Toll/interleukin-1 receptor (TIR-1) and generation of 5-hydroxytryptamine (5-HT) [33]. The discovery substantiates the finding that the immune response to pathogenic or nutritional bacteria in *C. elegans* needs a signaling pathway that contains the mammalian orthologs of Toll-interleukin-1 receptor (TIR) domain protein sterile *α* and TIR motif (SARM), the mitogen-activated protein kinase kinase kinase (MAPKKK) apoptosis signal-regulating kinase 1 (ASK1), and the mitogen-activated protein kinase kinase (MAPKK) MKK3, which activates p38 MAPK. The SARM-ASK1-MKK3 pathway functions in *C. elegans* in response to pathogens both in the cell-autonomous regulation of innate immunity and the neuroendocrine regulation of serotonin-dependent aversive behavior [34]. Aside from the previous mechanisms, *C. elegans* can adjust its olfactory preferences after exposure to pathogenic bacteria, avoiding odors from the pathogens and increasing its attraction to odors from familiar nonpathogenic bacteria [35].

 Next, the *C. elegans* transcription factor SKN-1, which regulates oxidative and xenobiotic stress response and profits longevity, is found to be necessary for pathogen resistance to both Gram-negative *Pseudomonas aeruginosa* and Gram-positive *Enterococcus faecalis*. Both the Toll-interleukin 1 receptor (TIR) domain containing adaptor protein TIR-1 and the p38 mitogen-activated protein kinase (MAPK) ortholog PMK-1 are needed in the SKN-1 stimulation by PA14 exposure [36]. Moreover, it has been observed that development of *C. elegans* larvae on pathogens results in enhanced resistance to different pathogens and to heat shock. The boosted pathogen resistance may be attributed to the early initiation of the heat shock response in the worms, and the resultant lifespan increase can be related to the DBL-1 transforming growth factor *β*-like (TGF*β*-like), DAF-2/DAF-16 insulin-like, and p38 MAP kinase pathways [37]. Also, c-Jun N-terminal kinase (JNK)-like MAPK but not p38 MAPK pathway is found as a key regulator of transcriptionally-induced pore-forming toxins (PFT) defenses, and the activator protein AP-1, a downstream target of the JNK-mediated PFT protection pathway, is uncovered as one of the cellular components important for protecting *C. elegans* against pathogen-produced PFTs [38].

In addition, *C. elegans* is capable of reducing the toxicity of various small molecule toxins produced by pathogens by chemically modifying O-/N-glycosylation and unusual 3′-O-phosphorylation of the resultant glucosides [39]. The characteristic antibiotic constituents such as antimicrobial peptides (AMPs) in *C. elegans* are essential to protect the worm against infection. The majority of AMPs in *C. elegans* are caenopores, and they kill bacteria by permeabilizing their cytoplasmic membrane and executing pore-forming activity. The AMPs are also required to tackle with the regular bacterial food of *C. elegans*, *Escherichia coli*, and without them, *C. elegans* grows poorly, with a great number of bacteria accumulated in the worm's intestine. Therefore, the caenopore class of AMP may be an important factor in enabling *C. elegans* to live with microbes [40]. Some AMP genes are induced upon contact with particular bacteria, whereas others are stimulated regardless of the bacteria they feed on. N-glycans are also found to play a role in the interaction of *C. elegans* with pathogenic bacteria, indicating that N-glycans are components of the worm's innate immune system [41]. Lysozymes are antimicrobial enzymes that play a pressing role in resisting infection in a wide scope of eukaryotes. Deletion of the protist type lysozyme LYS-7 can make *C. elegans* susceptible to killing by fungus *Cryptococcus neoformans*, a fatal human pathogen, but enhance its tolerance to the enteric bacteria *Salmonella Typhimurium*. These compound responses indicate higher levels of complexity in the *C. elegans* innate immune system [42]. *C. elegans* can respond to pathogens by generating reactive oxygen species (ROS) in the intestine while synchronically instigating an oxidative stress response which is dependent on transcriptional regulator DAF-16 to protect adjacent tissues [43]. 

Besides, it has been discovered that *C. elegans* heterochromatin protein HPL-1 interacts with the linker histone variant HIS-24 monomethylated at lysine 14 (HIS-24K14me1) and associates with promoters of the genes involved in the worm's antimicrobial response, suggesting a functional partnership between epigenetic regulation and the innate immune system in *C. elegans* [44]. Natural variation in the *C. elegans* resistance to pathogen infection has proven to be caused by a polymorphism in the NPR-1 gene, which encodes a mammalian neuropeptide Y receptor homolog. The NPR-1-mediated pathogen resistance mechanism, however, is via oxygen-dependent behavioral avoidance instead of direct regulation of innate immunity [45]. Another antimicrobial mechanism in *C. elegans* is the worm's antiviral RNA interference machinery, which has been discovered to function in dealing with the replication of the artificially introduced *Flock house virus* (FHV) and Orsay virus infection in *C. elegans* [7, 46].

The interaction between microbial pathogens and *C. elegans* is a relatively new research topic. It is likely that, apart from the previous means, other mechanisms of interaction between the pathogens and the worm have yet to be discovered. In particular, more research is needed to determine the complex interplay among different mechanisms.

## 3. Interactions between Commensal Microbes and *C. elegans*

### 3.1. Bacteria Are the Food Source of *C. elegans*

In the natural condition, *C. elegans* often encounters many different types of materials, and it uses mechanoreceptor neurons (MRNs) to recognize collisions with particles and detect bacteria. Hermaphrodites and males possess 22 putative MRNs; males possess another 46 MRNs, most, if not all, of which are needed for mating [47]. In the process of touch sensation, a mechanical stimulus is converted into electrical signals through the activation of ion channels that respond to mechanical stimuli [48]. Serotonin, an endogenous pharyngeal pumping activator whose action is triggered by bacteria, activates pharyngeal pumping and isthmus peristalsis (both are required motions in the process of *C. elegans*' feeding) by activating two separate neural pathways [49–51]. For activating pumping, the SER-7 serotonin receptor in the MC motor neurons in the feeding organ activates cholinergic transmission from MC to the pharyngeal muscles by triggering the Gs alpha signaling pathway. For activating isthmus peristalsis, the SER-7 in the M4 (and possibly M2) motor neuron in the feeding organ stimulates the G(12)alpha signaling pathway in a cell-autonomous manner, which probably initiates neurotransmission from M4 to the pharyngeal muscles [52]. Some soil bacteria are commensal, but others may be pathogenic. *C. elegans* is attracted to nutritious bacteria and is repelled by pathogens and toxins using mechanisms previously discussed. *C. elegans* discriminates food both physically, based on size, and chemically, based on taste and olfaction [53]. Interestingly, depending on its experience, *C. elegans* can also differentiate beneficial bacteria from toxic bacteria by increasingly releasing the neurotransmitter serotonin onto interneurons [54]. *C. elegans* has evolved diverse actions for seeking high quality food and leaving low quality bacteria. This food-searching performance is enhanced in the worms that have already experienced good food. When searching for good food, worms switch between two motion modes: dwelling, which is a kind of movement with recurrent ceases and turns which is common when animals are on good food, and roaming, which is a type of unswerving fast movement which usually happens when worms are on bad food. AIY neuron has been found serving to extend roaming terms, and it is capital for seeking effective food [55]. 

### 3.2. Bacterial Strain Affects the Life Span of *C. elegans*

Two strains of *Bacillus mycoides* and *Bacillus soli* have been singled out as *C. elegans*' preferred food compared to strain *E. coli* OP50, a common food used for the worm at laboratory, and both can extend the life span of *C. elegans* by activating the autophagic process [56]. *Lactobacillus rhamnosus* CNCM I-3690 also can lengthen the worm's life expectancy and induce different expression of the DAF-16/insulin-like pathway, which is greatly conserved in humans. Importantly, this may suggest that *C. elegans* can probably be used to identify new potential antioxidant probiotic strains for future use in humans [14]. Also, it has been observed that food limitation can increase the lifespan of *C. elegans* [57]. Additionally, as worms age, bacteria accumulate in the intestinal tract [58], and *C. elegans*' intestinal bacterial accumulation which is regulated by intestinal immunity is one of the important lifespan determinants (other factors are bacterial strain, worm genotype, and biologic age) [59].

### 3.3. Interspecies Signaling Occurs between *C. elegans* and Microbes

Bacterially derived nitric oxide (NO) produced from the natural food of *C. elegans*, Bacilli that contain functional NO synthase, is an essential signaling molecule in multicellular organisms. This compound elevates *C. elegans*' longevity and stresses resistance through a class of genes that act under the paired control of HSF-1 and DAF-16 transcription factors [60]. Moreover, a recent study reports that two *Escherichia coli* endogenous noncoding RNAs, OxyS and DsrA, affect *C. elegans* physiology. OxyS down regulates che-2, leading to *C. elegans* chemosensory behavior impairment, and DsrA inhibits diacylglycerol lipase gene F42G9.6, giving rise to a longevity decrease. The study indicates that noncoding RNAs might have interspecies ecological roles [61]. Another study [62] finds that bacterial strain affects the fat storage of *C. elegans* through interspecies signaling. The authors have reported that nutrient differences of fatty acid composition and carbohydrate levels in different *E. coli* strains were not found to have a causative effect on fat storage levels in the worms; instead, specific peptides or amino acids may have provided nutritional signals regulating fat storage levels.

### 3.4. *C. elegans*' Behavioral State Varies in Response to Bacterial Supplies

Food quantity and quality can modulate *C. elegans*' behavioral stages. If worms are given sufficient high-quality food, they will finally become saturated and stagnant and stop eating and moving [63]. If the first larval stage (L1) worms are starved, they can choose to enter into dauer stage in which *C. elegans* becomes thin, dense, and motionless unless disturbed, probably for reserving energy. Dauer larvae usually gather together into a droplet of condensation. Another dauer-specific behavior is nictation in which the larva sets a projection and jumps up on its tail, swaying its head in the air [64]. This would possibly allow the dauer larva to adhere to passing organisms to be transferred itself to a new environment or to search for food source. Dauer larvae can survive several months without food. After the food is provided again, they can bypass the larval stages 2 (L2) and 3 (L3) and get into the fourth larval stage (L4) directly [65].

## 4. Interactions between Host and Intestinal Microbiota in *C. elegans*

As far as we are aware, all the investigations on host-microbe in *C. elegans* had dealt with only one or few bacterial species till the present year. In 2013, Shapira's research group first characterized the *C. elegans* natural gut microbial communities by growing germ-free worms in a natural-mimic environment of soil and rotting fruit, with 18 species having been identified. These species include *Bacillus *sp., *Bacillus megaterium/Bacillus *sp.*/Bacillus aryabhattai/Bacillus thuringiensis*, *Bacillus foraminis/Bacillus asahii*, *Bacillus nealsonii/Bacillus circulans*, *Bacillus subtilis*, *Paenibacillus *sp.*/Paenibacillus lautus/Paenibacillus odorifer*, *Lysinibacillus fusiformis/Lysinibacillus xylanilyticus/Lysinibacillus sphaericus*, *Staphylococcus warneri/Staphylococcus pasteuri*, *Bacillus *sp.*/Bacillus pumilus*, *Pseudomonas *sp.*/Pseudomonas oleovorans/Pseudomonas stutzeri/P. mendocina/Pseudomonas pseudoalcaligenes*, *P. aeruginosa/P. mendocina/Pseudomonas alcaligenes*, *P. oleovorans/P. mendocina/P. pseudoalcaligenes*, *Pseudomonas trivialis/Pseudomonas poae*, *Pseudomonas fluorescens/Pseudomonas moraviensis/Pseudomonas koreensis/Pseudomonas putida*, *Arthrobacter *sp., *Enterobacter *sp.*/Pasteurella aerogenes*, *Rahnella aquatilis*, and *Buttiauxella agrestis/Buttiauxella noackiae*. They discovered that members of *C. elegans* microbiota can enhance the host's pathogenic resistance, although disparate components may employ distinct resistance mechanisms. For the two primary species of the identified bacteria, *Bacillus megaterium *(BM) and *Pseudomonas mendocina *(PM), the increase in host's infection resistance supplied by BM is related to the compromised reproduction, while the protection provided by PM is reproduction-independent, by activating the p38-dependent pathway to prime the worm immune system. Considering the p38 pathway is one of the functionally conserved signaling pathways throughout the animal kingdom, this disclosure may indicate that similar interactions might also be present in mammals [66].

## 5. Outlook

It has been well recognized that mammals, including humans, have highly complex microbiota in their intestines that contributes to gut maturation, host nutrition, and pathogen resistance as well as regulate host energy metabolism and inflammatory immune responses. Therefore, a good understanding of intestinal microbial ecology allows us to better manage and promote human health. Due to the difficulty of using human subjects as models in experiments, mice have been commonly used in the ecological study of mammal intestinal microbial communities; however, the generation interval of mice is long, and it is more difficult and costly to handle the animal. Given the advantages of *C. elegans* as a model animal and importantly the lower costs associated with *C. elegans *experiments, an interesting question to ask is the following: can studying the host-microbiota interactions in *C. elegans* confer insights into the counterparts in mammals? At the moment, there is no clear answer to it. 

*C. elegans* has been widely used as a model to investigate animal development and behavior, but the research on interaction between microbe and *C. elegans* is still relatively new. Recent investigations have greatly enhanced our understanding of *C. elegans*-microbe relationships, but all except one involve only one or few microbial species. More research is needed to examine, for instance, how a complex microbial community may evolve and be established in the worm's intestine and how this microbiota interacts with the host before we can confidently answer the previous question.
